# Measurement of intracranial pressure and short-term outcomes of patients with traumatic brain injury: a propensity-matched analysis

**DOI:** 10.5935/0103-507X.20150055

**Published:** 2015

**Authors:** Cesar Biselli Ferreira, Estevão Bassi, Lucas Lucena, Hernandez Carreta, Leandro Costa Miranda, Paulo Fernando Guimarães Mazorcchi Tierno, Robson Luis Amorim, Fernando Godinho Zampieri, Luis Marcelo Sá Malbouisson

**Affiliations:** 1 Trauma Intensive Care Unit, Hospital das Clínicas, Faculdade de Medicina, Universidade de São Paulo - São Paulo (SP), Brazil.; 2 Intensive Care Unit, Hospital Alemão Oswaldo Cruz - São Paulo (SP), Brazil.; 3 Discipline of Neurosurgery, Universidade de São Paulo - São Paulo (SP), Brazil.; 4 Intensive Care Unit, Discipline of Emergency Medicine, Hospital das Clínicas, Faculdade de Medicina, Universidade de São Paulo - São Paulo (SP), Brazil.

**Keywords:** Brain injuries, Intracranial pressure monitoring

## Abstract

**Objective:**

To assess the impact of intracranial pressure monitoring on the short-term outcomes of traumatic brain injury patients.

**Methods:**

Retrospective observational study including 299 consecutive patients admitted due to traumatic brain injury from January 2011 through July 2012 at a Level 1 trauma center in São Paulo, Brazil. Patients were categorized in two groups according to the measurement of intracranial pressure (measured intracranial pressure and non-measured intracranial pressure groups). We applied a propensity-matched analysis to adjust for possible confounders (variables contained in the Crash Score prognostic algorithm).

**Results:**

Global mortality at 14 days (16%) was equal to that observed in high-income countries in the CRASH Study and was better than expected based on the CRASH calculator score (20.6%), with a standardized mortality ratio of 0.77. A total of 28 patients received intracranial pressure monitoring (measured intracranial pressure group), of whom 26 were paired in a 1:1 fashion with patients from the non-measured intracranial pressure group. There was no improvement in the measured intracranial pressure group compared to the non-measured intracranial pressure group regarding hospital mortality, 14-day mortality, or combined hospital and chronic care facility mortality. Survival up to 14 days was also similar between groups.

**Conclusion:**

Patients receiving intracranial pressure monitoring tend to have more severe traumatic brain injuries. However, after adjusting for multiple confounders using propensity scoring, no benefits in terms of survival were observed among intracranial pressure-monitored patients and those managed with a systematic clinical protocol.

## Abstract (pt)

**Objetivo:**

Avaliar o impacto do monitoramento da pressão intracraniana nos desfechos em curto prazo de pacientes com lesão encefálica traumática.

**Métodos:**

Estudo retrospectivo e observacional que incluiu 299 pacientes consecutivos admitidos por lesão cerebral traumática entre janeiro de 2011 e julho de 2012 em um centro de trauma Nível 1 localizado em São Paulo (SP). Os pacientes foram categorizados em dois grupos, segundo a mensuração da pressão intracraniana (grupos com mensuração da pressão intracraniana e sem mensuração da pressão intracraniana). Aplicamos uma análise de propensão pareada para ajustar quanto a possíveis fatores de confusão (variáveis contidas no algoritmo prognóstico CRASH Score).

**Resultados:**

A mortalidade global aos 14 dias (16%) foi equivalente à observada em países desenvolvidos no estudo CRASH, e melhor que o previsto com base na calculadora de escore CRASH (20,6%), com uma proporção padronizada de mortalidade de 0,77. No total, 28 pacientes receberam monitoramento da pressão intracraniana (grupo com mensuração da pressão intracraniana), dos quais 26 foram pareados em proporção 1:1 com pacientes do grupo sem mensuração da pressão intracraniana. Não houve melhora no grupo com mensuração da pressão intracraniana em comparação àquele sem mensuração da pressão intracraniana quanto à mortalidade hospitalar, à mortalidade aos 14 dias, ou à mortalidade combinada hospitalar e em hospital de retaguarda. A sobrevivência até 14 dias foi também similar entre os grupos.

**Conclusão:**

Os pacientes que receberam monitoramento da pressão intracraniana tendem a ser portadores de lesões encefálicas mais graves. Porém, após ajustar quanto a múltiplos fatores de confusão com a utilização de um escore de propensão, não se observou qualquer benefício em termos de sobrevivência entre os pacientes com monitoramento da pressão intracraniana em relação aos tradados segundo um protocolo clínico sistematizado.

## INTRODUCTION

Traumatic brain injury (TBI) is a medical and social problem worldwide, with an estimated 10 million cases leading to hospitalization or death each year.^(1)^ The worldwide incidence of TBI is increasing, especially in developing countries where the use of motor transportation is also increasing.^(2)^ Despite improvements in trauma systems and critical care, mortality rates of approximately 40% have been reported in a review of unselected observational studies.^(3)^ In addition to death, disability in young, productive people leads to large direct and indirect costs to society.^(2)^

The Brain Trauma Foundation recommends monitoring intracranial pressure (ICP) in all patients with survivable severe traumatic brain injuries and abnormalities observed on a computed tomography (CT) scan obtained at the time of admission as well as in selected patients (e.g., those who are over the age of 40 years with hypotension or abnormal flexion or extension in response to pain) with a normal CT scan.^(4)^ The insertion of an intracranial catheter carries risks of hemorrhage and infection, and the benefits of such monitoring in patients with TBI have been questioned in recent years.^(5)^

Chesnut et al. recently conduct a clinical trial to determine the potential benefits of monitoring ICP. They found no benefit of directly measuring ICP compared to patients managed with a rigorous and aggressive clinical protocol to control intracranial pressure.^(6)^ However, there were caveats in the pre-hospital care in the Chesnut´s study, such as a low proportion of patients admitted to the hospital by ambulance; therefore, the impact of an aggressive strategy of ICP monitoring may have been mitigated.

We sought to compare two groups of TBI management (with an ICP monitor and with a systematic clinical approach to control ICP without an invasive monitor) in a context outside of a clinical trial in a trauma population in a middle-income country.

## METHODS

This retrospective observational study included 299 consecutive patients admitted due to traumatic brain injury from January 2011 through July 2012 at a Level 1 trauma center in São Paulo, Brazil. Patients were categorized in two groups according to the measuring of ICP (measured ICP and non-measured ICP groups). All data were collected during ICU admission using a standardized database system. Demographic and clinical data were collected during intensive care unit (ICU) admission, and patients were followed-up until hospital discharge. Discharge to a chronic care facility was also noted. This study was approved by the Research Ethics Committee of the *Hospital das Clínicas* of the *Faculdade de Medicina* of the *Universidade de São Paulo* (CAPPesq, approval number 279.097). Informed consent was waived due to the strict observational nature of the study.

Head CT were evaluated independently by two investigators (CBF and EB) and classified according to the components of the Crash prognostic score system. Disagreements between the two investigators were resolved by a third investigator (LCM). The following characteristics of the head CT were evaluated: presence of midline shift, presence of subarachnoid hemorrhage, presence of non-evacuated hematoma, presence of petechia, and obliteration of the third ventricle. We also observed whether the patient had a major extracranial injury; the first available Glasgow coma scale score (GCS, at the trauma scene if available or at hospital admission); and pupil reaction to light (both, one or none).

An intracranial pressure catheter was inserted at admission at the discretion of the attending neurosurgeon. When the patient had already been admitted to the ICU, the indication was discussed with the intensivist. Of the patients in the study, 28 received ICP monitoring (measured ICP group), with 11 of those receiving an external ventricular drainage system and the other 17 receiving an intraparenchymal device (Raumedic^®^, Helmbrechts, Germany). Intraparenchymal probes were calibrated in the operating room. When an external ventricular drainage system was placed, it was zeroed at the tragus level (as was the invasive arterial line for arterial blood pressure monitoring). Cerebral perfusion pressure was calculated as ICP minus mean arterial blood pressure.

### Management of traumatic brain injury for patients in the measured intracranial pressure group

Patients in the measured ICP group had therapy targeted to maintain ICP ≤ 20mmHg and cerebral perfusion pressure between 60 and 70mmHg. ICP control was initially attempted through the use of continuous sedative agents (propofol and fentanyl), 30º head positioning; hyperosmolar therapy (0.5mL/kg bolus of NaCl 20% and NaCl 3% as maintenance aimed at a serum sodium level between 145 and 150mEq/L); and normocapnia (arterial partial pressure of CO_2_ - PaCO_2_ - between 35 and 38mmHg). If an external ventricular drainage system was in place, it was left open with a starting drainage pressure of 20mmHg. Second-tier therapies included induced hypothermia (using cooling blankets aimed at a central temperature between 34 and 35°C); barbiturates (thiopental, 3 - 5mg/kg/h until burst suppression on electroencephalography); and, in selected cases, mild hyperventilation (target PaCO_2_ between 28 and 30mmHg). A decompressive craniectomy was used as a last resource in selected cases. Therapies were weaned from the most recently added to the initial therapies according to the reduction in ICP levels.

If the third ventricle was obliterated at admission, patients were kept sedated with propofol guided by EEG (targeting burst-suppression). A head CT was repeated within 24 hours post-trauma and then every 48 hours or if there was a new critical event (such as anisocoria or an increased pulsatility index in transcranial Doppler - TCD). TCD was performed on a daily basis and acted as an adjuvant tool to orient the therapy. All patients were managed with continuous hypertonic saline infusion (NaCl 3%, titrated to a mean serum sodium level of 150mEq/L). Second-tier therapies were added on a case-by-case analysis only if the head CT showed no improvement during the initial 48 hours after ICU admission. Sedatives and hypertonic saline infusions were withheld when the head CT showed an open third ventricle.

Corticosteroids were not used in TBI patients, as supported by major recommendations. Prophylactic anticonvulsants (phenytoin, 100mg every eight hours) were used as recommend. Glycemic control was achieved through intravenous insulin adjusted by point-of-care measurements targeting a blood glucose level between 150 and 200mg/dL.

Our primary endpoint was hospital mortality. As a secondary endpoint, we evaluated 14-day mortality, discharge to chronic care facility, and combined mortality (hospital plus chronic care facility mortality). The latter analyses were possible because the chronic care facility that receives patients from our hospital is linked to our institution (*Hospital Auxiliar de Suzano*, Suzano, Brazil) and shares the same electronic database.

### Statistical analysis

Continuous variables were tested for normality using the Kolmogorov-Smirnov test. Parametric continuous variables were compared between groups using Student's *t* test. Non-parametric continuous variables were compared using the Mann-Whitney test. Categorical variables and dichotomic outcomes were compared by the Chi square test (with Yates correction when indicated) or Fisher´s exact test. We expected that patients who received ICP monitoring would differ from patients who did not receive ICP monitoring in several ways. Therefore, to adjust for confounders, we used a propensity-matched analysis that would adjust for possible confounders. The propensity score (PS) resembled the given probability of a patient receiving a specific therapy. We defined a priori that the PS would be adjusted for all variables contained in the Crash Score prognostic algorithm, namely age; gender; GCS; pupil reaction to light; major extracranial injury; and head CT features, including presence of non-evacuated hematoma, midline shift, subarachnoid hemorrhage, and obliteration of third ventricle. The PS was obtained using logistic regression with ICP measurement as the outcome. Matching was performed using a 1:1 basis (one patient who received ICP monitoring for one who did not) using a caliper of 0.05. The 14-day survival rate for matched patients was also assessed by a Kaplan-Meier plot with a log rank test.

All analyses were conducted using R project with matching and survival packages.

## RESULTS

The -day mortality rate of the total sample was 16%, which was very similar to what was expected based on the CRASH score (20.6%) for high-income countries, with a standardized mortality ratio of 0.77. Of 299 patients included in the analysis, only 28 (9.3%) received ICP monitoring during their ICU stay. Characteristics of the whole population and comparisons between patients with and without ICP monitoring are shown in table 1. In brief, most patients were male (83%) and young (mean age of 39 years old, range 13 - 90 years). Patients in the measured ICP group had lower GCS scores at the scene and more frequently had subarachnoid hemorrhage. Without adjustment for confounders, patients in the measured ICP group had a longer ICU length of stay (LOS) and a higher mortality rate (both in-hospital and combined in-hospital plus chronic care facility mortality - Table 1).

**Table 1:** Overall characteristics of the whole population and comparisons between patients in the measured intracranial pressure and the non-measured intracranial pressure groups

Demographic features and characteristics	All patients	Measured ICP	Non-measured ICP	p value
	(N = 299)	(N = 28)	(N = 271)	
Age	39 [28 - 53]	39.4 [23.5 - 47]	39 [28 - 53]	0.282
Gender, male	248 (83)	21 (75)	227 (83)	0.240
GCS	8 [5 - 13]	6.5 [3 - 8]	9 [5 - 13]	0.004
Method for ICP measurement				
Intraventricular catheter	11 (3.6)	11 (39)	-	-
Intraparenchimatous probe	17 (5.6)	17 (61)	-	-
One pupil does not react	73 (24.4)	8 (28.5)	65 (23.9)	0.590
Two pupils do not react	33 (11)	2 (7.1)	31 (11.4)	0.489
Major extracranial injury	145 (48.4)	12 (42.8)	133 (49.0)	0.530
Petechia on head CT	87 (29.0)	12 (42.8)	75 (27.6)	0.092
Obliterated third ventricle	40 (13.3)	4 (14.2)	36 (13.2)	0.882
Subarachnoid hemorrhage	199 (66.5)	28 (100)	171 (63)	< 0.001
Midline shift	74 (24.7)	9 (32.1)	65 (23.9)	0.341
Non-evacuated hematoma	61 (20.4)	8 (28.5)	53 (19.5)	0.259
Outcomes				
ICU length of stay (days)	8 [3 - 15]	10 [6.75 - 16.25]	7 [3 - 15]	0.030
Hospital survivor ICU LOS	9 [3 - 16]	14 [9 - 23]	8 [3 - 15.25]	0.037
Hospital length of stay (days)	16 [7 - 27]	17 [7 - 27]	16 [7 - 28]	0.750
Hospital survivor hospital LOS	18 [9 - 30]	19 [16 - 27]	18 [9 - 31.25]	0.559
Discharge to chronic care facility	93 (31.1)	12 (42.8)	81 (29.8)	0.158
14-day mortality	48 (16)	8 (30)	40 (14)	0.104
Hospital mortality	62 (20.7)	11 (39.2)	51 (18.8)	0.010
Combined hospital and chronic care facility mortality	80 (26.7)	14 (50)	66 (24.3)	0.003

ICP - intracranial pressure; GCS - Glasgow coma scale; CT - computed tomography; ICU - intensive care unit; LOS - length of stay. The results are expressed as the number (%) or median [25% - 75%].

**Figure 1:**
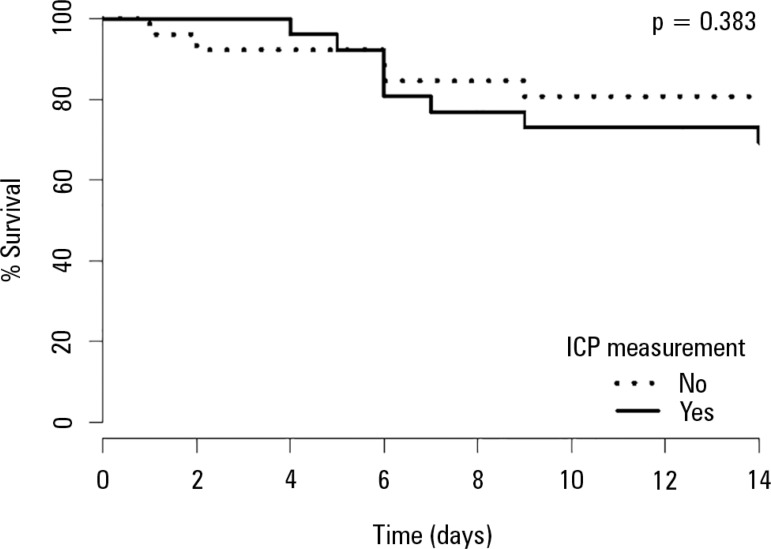
Kaplan -Meier plot ICP - intracranial pressure.

Propensity score matching pairing was possible for 26 of the 28 patients in the measured ICP group. The results comparing propensity-matched patients in the measured ICP and non-measured ICP groups are shown in table 2. Propensity score matching allowed the creation of two groups that had similar characteristics for all components of the Crash score. The ICU length-of-stay among survivors was higher in the measured ICP group (16 [9.5 - 24.5] versus 10 [4 - 14] in the non-measured ICP group; p = 0.047). There was no significant difference between groups in mortality outcomes. Survival up to 14 days was similar between both matched groups (Figure 1). Frequency of discharge to a chronic care facility was similar between groups.

**Table 2:** Comparison between matched patients in the measured intracranial pressure and non-measured intracranial pressure groups

	Measured ICP	Non-measured ICP	p value
	(N= 26)	(N = 26)	
Age	36 [24.25 - 44]	31.5 [22 - 49.5]	0.783
Male	23 (88)	21 (80)	0.700
GCS	6.5 [3 - 8]	5 [3 - 7]	0.397
One pupil does not react	7 (27)	5 (19)	0.742
Two pupils do not react	2 (8)	2 (8)	1
Major extracranial injury	12 (50)	12 (50)	1
Petechia on head CT	10 (39)	7 (27)	0.554
Obliterated third ventricle	4 (15)	3 (11)	0.684
Subarachnoid hemorrhage	26 (100)	26 (100)	1
Midline shift	8 (31)	7 (27)	0.755
Non-evacuated hematoma	7 (27)	7 (27)	1
Outcomes			
ICU length of stay (days)	10 [7 - 16.75]	7 [3.25 - 14]	0.09
Hospital survivor ICU LOS	16 [9.5 - 24.5]	10 [4 - 14]	0.047
Hospital length of stay (days)	17 [7 - 27]	14.5 [7.25 - 21]	0.359
Hospital survivor hospital LOS	19 [16.5 - 27]	17 [10 - 22]	0.193
Discharge to chronic care facility	10 (38)	9 (35)	0.773
14-day mortality	8 (30)	4 (15)	0.323
Hospital mortality	11 (42)	5 (19)	0.133
Combined hospital and chronic care facility mortality	13 (50)	7 (27)	0.154

ICP - intracranial pressure; GCS - Glasgow coma scale; CT - computed tomography; ICU - intensive care unit; LOS - length of stay. The results are expressed as the number (%) or median [25% - 75%].

## DISCUSSION

This study showed no clear evidence that ICP monitoring offers any short-term survival advantages in TBI patients. In a propensity score analysis adjusted for major short-term confounders, mortality, survival up to 14 days and frequency of discharge to chronic care facilities were not different between groups. Global mortality in 14 days (16%) was equal to that observed in high-income countries in the CRASH Study and was better than expected based on the CRASH calculator.^(7)^

The association between intracranial hypertension and poorer outcomes in TBI patients has been known since 1951.^(8)^ Evidence in favor of strict intracranial pressure control gained importance during the following years, and it is currently recommended by international guidelines.^(4,5,9)^ While direct pressure monitoring is the gold standard for ICP measurement and clinical management, its use is associated with risks, such as bleeding and infection, that may overcome the benefits of ICP control. Data comparing management strategies with and without ICP monitoring has yielded conflicting results.^(10-13)^ Chesnut et al. performed a randomized controlled trial assessing the role of ICP measurement in 324 TBI patients and concluded that ICP monitoring offered no advantages in terms of both short- and long-term mortality.^(6)^ Nevertheless, major concerns regarding pre-hospital care may have mitigated the potential benefit of ICP measurement. Therefore, the role of ICP monitoring in scenarios where pre-hospital care is structured and performed by trained personnel may be different.^(14,15)^ All patients included in our analysis were transported to the hospital by helicopter or ground ambulance by trained personnel.

ICP monitor use is highly variable.^(16,17)^ Despite its use being widely accepted, some reports suggest that close to 20% of neurosurgeons have a high level of confidence that ICP monitoring improves prognosis in TBI patients.^(17)^ It could be suggested that our results could be related to the lack of experience of the ICU personnel in dealing with neurotrauma patients; however, our expected short-term mortality was proven to be similar to that of high-income countries when using the CRASH model. Our results are aligned with not only the most recent randomized controlled trial but also a recent meta-analysis on this subject.^(18)^ It should be highlighted that from the six studies that favored ICP monitoring included in this recent meta-analysis, five were conducted in developed countries.

There are several limitations in the present study. We could not account for all possible confounders in this analysis, such as other markers of illness severity. We do not have data regarding pre-hospital care, the specific number of interventions each patient received for ICP control, and the results from monitoring devices (for example, individual transcranial Doppler data or ICP values). Despite the moderate size of our sample, few patients received ICP monitoring. Nevertheless, we were able to adequately pair most (26 out of 28) patients in the monitored ICP group with non-monitored ICP group patients using a propensity score analysis, which may produce results that are similar to those of randomized controlled trials.^(19)^ The retrospective nature of the study leads to its inherent weaknesses, such as reporting bias; however, most of the data collection was performed using a computerized databank. Moreover, this single-center study was limited to bias in local care. Beyond that, other relevant outcomes of TBI, such as quality of life and long-term mortality, were not assessed. Therefore, our conclusions should be interpreted with caution, considering the specific features of the center where this study took place.

## CONCLUSION

In a cohort of traumatic brain injury patients, intracranial pressure monitoring was mainly used for patients with more severe traumatic brain injuries. After adjusting for multiple confounders using propensity score matching, no differences in terms of survival were observed among intracranial pressure-monitored patients compared to those managed with a systematic clinical protocol.
